# Entire Sound Representations Are Time-Compressed in Sensory Memory: Evidence from MMN

**DOI:** 10.3389/fnins.2016.00347

**Published:** 2016-07-26

**Authors:** Seiji Tamakoshi, Nanako Minoura, Jun'ichi Katayama, Akihiro Yagi

**Affiliations:** ^1^Department of Psychology, Tezukayama Gakuin UniversitySakai, Japan; ^2^Department of Psychological Science, Kwansei Gakuin UniversityNishinomiya, Japan

**Keywords:** event-related brain potential, mismatch negativity, sound representations, time-compression, temporal window of integration

## Abstract

In order to examine the encoding of partial silence included in a sound stimulus in neural representation, time flow of the sound representations was investigated using mismatch negativity (MMN), an ERP component that reflects neural representation in auditory sensory memory. Previous work suggested that time flow of auditory stimuli is compressed in neural representations. The stimuli used were a full-stimulus of 170 ms duration, an early-gap stimulus with silence for a 20–50 ms segment (i.e., an omitted segment), and a late-gap stimulus with an omitted segment of 110–140 ms. Peak MMNm latencies from oddball sequences of these stimuli, with a 500 ms SOA, did not reflect time point of the physical gap, suggesting that temporal information can be compressed in sensory memory. However, it was not clear whether the whole stimulus duration or only the omitted segment duration is compressed. Thus, stimuli were used in which the gap was replaced by a tone segment with a 1/4 sound pressure level (filled), as well as the gap stimuli. Combinations of full-stimuli and one of four gapped or filled stimuli (i.e., early gap, late gap, early filled, and late filled) were presented in an oddball sequence (85 vs. 15%). If compression occurs only for the gap duration, MMN latency for filled stimuli should show a different pattern from those for gap stimuli. MMN latencies for the filled conditions showed the same pattern as those for the gap conditions, indicating that the whole stimulus duration rather than only gap duration is compressed in sensory memory neural representation. These results suggest that temporal aspects of silence are encoded in the same manner as physical sound.

## Introduction

In order to process continuously changing auditory information over the course of everyday life, the human brain integrates auditory information as a unitary event about every 200 ms. This auditory integration duration is called a temporal window of integration (TWI), and it is used to integrate processes as a “sliding window” in the sound representation (Näätänen, 1990, 1992; Näätänen and Winkler, 1999; Näätänen et al., 2007). Previous behavioral studies have shown a relationship between the TWI and auditory phenomena such as loudness summation and backward recognition masking. Loudness summation, i.e., the increased sound loudness with the sound duration, is observed up to 200 ms (Moore, 1989). In addition, the backward recognition masking effect is observed when stimuli are closely presented within 250 ms interval (Massaro, 1972). These effects are indicating that the integration process was performed in the temporal window about 200 ms (Cowan, 1984; Moore, 2003).

Several studies have investigated the TWI using mismatch negativity (MMN), which is an event-related brain potential (ERP) component that reflects pre-attentive auditory processing. MMN is elicited when stimuli violate the regularity of preceding stimuli (regularity in pitch, intensity, duration, or a combination of these features; Winkler et al., 2003; Näätänen et al., 2005, 2007; Winkler, 2007). Regularity is formed by preceding repetitive stimuli, called the “standard” in the commonly used oddball sequence paradigm. Some studies have measured MMN to investigate TWI, using the backward recognition masking paradigm (Winkler and Näätänen, 1992), stimulus omission paradigm (Yabe et al., 1997, 1999), stimulus omission and loudness summation (Oceák et al., 2006), with pairs of two closely spaced tones (Tervaniemi et al., 1994), a successive double deviants paradigm (Sussman et al., 1999; Wang et al., 2005), or by using complex sounds formed by combining two different features in the middle of the stimulus (Winkler et al., 1998; Grimm and Schröger, 2005). These studies all show that closely presented sounds are integrated within the TWI that is estimated with the duration about 170 ms or slightly longer.

However, little is known about time flow in sensory memory representation, as integrated within the TWI. To elucidate such as temporal aspect in the representation leads to understanding our integrative or objective processing in the continuously changing auditory information. The process of temporal integration is thought that an input stimulus is analyzed for each stimulus feature, after then the information of different features to be integrated as a unitary stimulus representation (Näätänen and Winkler, 1999). According to this proposal, the time course within the TWI does not correspond to real time-flow (Näätänen, 1990; Näätänen et al., 2007). One previous study supported this hypothesis by showing that the time flow of memory representation is indeed compressed (Yabe et al., 2005). Yabe et al. (2005) used three auditory stimuli. One was a continuous tone of 170 ms duration (full-stimulus), and the others included a silent segment (i.e., a partial omission or gap) at either early or late time points in the tone. Continuous and gap stimuli were paired in an oddball sequence. These researchers showed that MMNm peak latencies changed asymmetrically when standard stimulus was switched to deviant stimulus. MMNm peak latencies reflected the time point of inserted gaps (i.e., shorter latencies for early gap stimuli and longer latencies for late gap stimuli), when full-stimuli were presented as standard. However, when reversing the role of the stimuli (i.e., full-stimuli were presented as deviant and gap-stimuli as standard), the difference in peak latency between early and late conditions was reduced (i.e., latencies for the early condition were prolonged whereas late condition latencies were reduced). The reduced latency in the late condition could not be explained if the time flow in the representation is the same as that in the real time. The authors interpreted these asymmetrical changes in MMN peak latencies as follows: because the MMNm peak latencies reflected timing of deviancy detection between deviant and standard sounds, the time flow in the neural representation may differ from that in the real world, such that time flow of sound with gaps showed a reduced duration in sensory memory. The researchers called this phenomenon “time compression.”

However, it remains unclear how exactly sound representations are compressed in sensory memory. Previous results showed that time compression occurs toward the center portion of the stimulus or the timing of inserted gaps (Yabe et al., 2005), because the latency difference between early and late time points was reduced. Thus, there are at least two possibilities: that only the silence portions of the stimuli or gap durations are compressed, or that the whole stimulus duration is compressed. In the case of the first possibility, a continuous sound (no silent gaps) is maintained in its original time scale. Indeed, compression of silence seems efficient, as silence contains no information.

In order to examine whether the whole stimulus or only gap duration is compressed, stimuli which included a 1/4 sound pressure level tone segment (filled stimulus) instead of a gap were used in this study. If only the gap duration is compressed, MMN latencies from a filled stimulus condition should show a different pattern from that of a gap stimulus condition. If the whole stimulus duration is compressed, there should be the same MMN latency pattern for filled and gap conditions.

## Methods

### Participants

Participants were 12 healthy adults reporting normal hearing (mean age 21.9 years, range 20–26 years, 6 males). The present study was conducted using a method approved by the Kwansei Gakuin University (KGU) Research Ethics Review Board, under the KGU Regulations for Research with Human Participants. Written informed consent was obtained from all participants and their rights as experimental participants were protected.

### Stimuli

**Figure 1:**
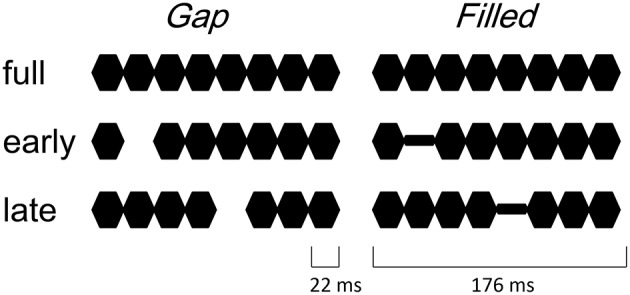
**Illustrated stimuli in the gap conditions (left panel) and filled conditions (right panel)**.

The procedure and stimulus parameters of this study followed previous work (Yabe et al., 2004, 2005). Stimuli were a train of sinusoidal tone segments presented at a 1000 Hz frequency (18 ms plateau and 2 ms rise / fall, see Figure 1). The full stimulus (full) consisted of 8 segments. The 2nd segment was omitted in early gap stimuli (early-gap), and the 5th segment was omitted in late gap stimuli (late-gap). The train duration was 176 ms and stimuli were presented through headphones at an intensity of 70 dB SL. In addition to the gap stimuli, early filled stimuli (early-filled) and late filled stimuli (late-filled) were created, in which gaps were replaced by a tone segment presented at 1/4 intensity.

This intensity was set during a pilot study according to the same level as that identified for hit rates in previous work (Yabe et al., 2005). Stimulus sequences consisted of two different stimuli (full and one of the other stimuli), at the standard with a probability of 85% and the other as deviant with a 15% probability. Stimulus onset asynchrony was 500 ms. Stimulus sequences were named like “deviant in standard,” with stimulus names of them. Four stimulus sequences were employed in the gap condition, i.e., early-gap in full, late-gap in full, full in early-gap, and full in late-gap. Similarly, in the filled condition, the following four stimulus sequences were utilized: Early-filled in full, late-filled in full, full in early-filled and full in late-filled. Stimulus sequences were presented in separate blocks. Order of these stimulus sequences was randomized across participants.

All data acquisitions were collected in an electrically shielded and sound-attenuated room. Participants underwent EEG recording and behavioral measurement on the same day; these were conducted in a counterbalanced order.

### EEG measurement session

During the EEG recording session, participants were required to watch a silent movie and ignore auditory stimuli. In each block, 800 stimuli were presented. EEG was recorded through Ag/AgCl electrodes using a SynAmps Model 5083 (Neurosoft Inc.), with a 1000 Hz sampling rate in the 0.05–100 Hz range from Fz, Cz, Pz, and right and left mastoids (10–20 system), with the nose tip as reference and AFz for the ground electrode. MMN analysis involved the EEG recorded from the Fz electrode, where the largest MMN is observed. Before calculating MMN, EEG was filtered offline using a 20 Hz high cut filter (24 dB/oct.). The analysis epoch was defined as 100 ms before and 350 ms after stimulus onset, with the former serving as a baseline. Epochs that contained potentials exceeding ± 80 μV on any channel were rejected. In order to cancel out the possible different effect of the different physical stimulus (i.e., the position of the gap or low-intensity segment) on the evoked potential, MMN to the deviants was calculated by subtracting waveforms of physically identical standard stimuli from those to deviant stimuli. For example, MMN for deviants in “early gap in full” was obtained by subtracting waveform of standard stimuli in “full in early gap” from that of deviant stimuli in “early gap in full.”

For the subtracted waveforms, MMN elicitation was defined as a significant difference with one-tailed Student's *t*-test compared to zero. MMN peaks were collected from each participant's waveform as the most negative point within ±50 ms from the peak point of the grand-averaged waveform. Statistical analysis was carried out using repeated measures analysis of variance (ANOVA).

### Behavioral measurement session

Reaction times (RTs) for behavioral discrimination of deviants were measured in a separate session. The participants were required to listen to the auditory stimulus sequence and to press a button as quickly as possible in response to the deviant stimulus. Stimulus onset asynchrony was again set at 500 ms. There were 300 stimuli in each block. RT was defined as the time between deviant onset and button pressing, not from the onset of the gap. Acceptable responses occurred 200 to 500 ms after deviant onset. Correct response rate was calculated as the number of acceptable responses divided by total number of deviants.

## Results

### MMN

**Figure 2:**
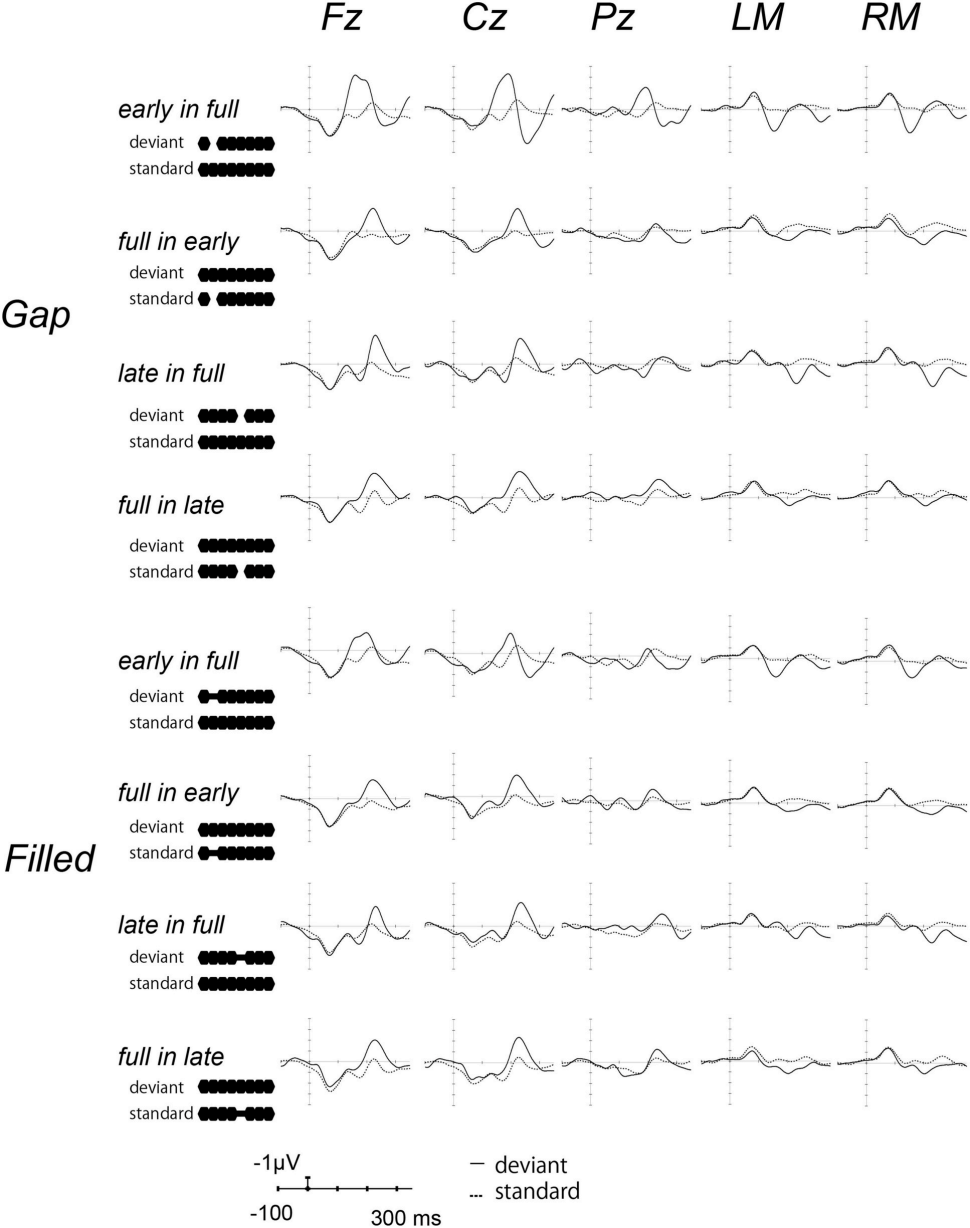
**Grand-averaged waves elicited by standard and deviant stimuli at each electrode site**. Upper panel shows the waves from the gap conditions, lower panel shows those for the filled conditions. The solid line represents ERPs to deviant stimuli, the dotted line ERPs to standard stimuli. For the presentation purpose, the waveforms were digitally low-pass filtered at 20 Hz (24 dB/octave) with a zero-phase filter, in this and the following waveforms.

**Figure 3:**
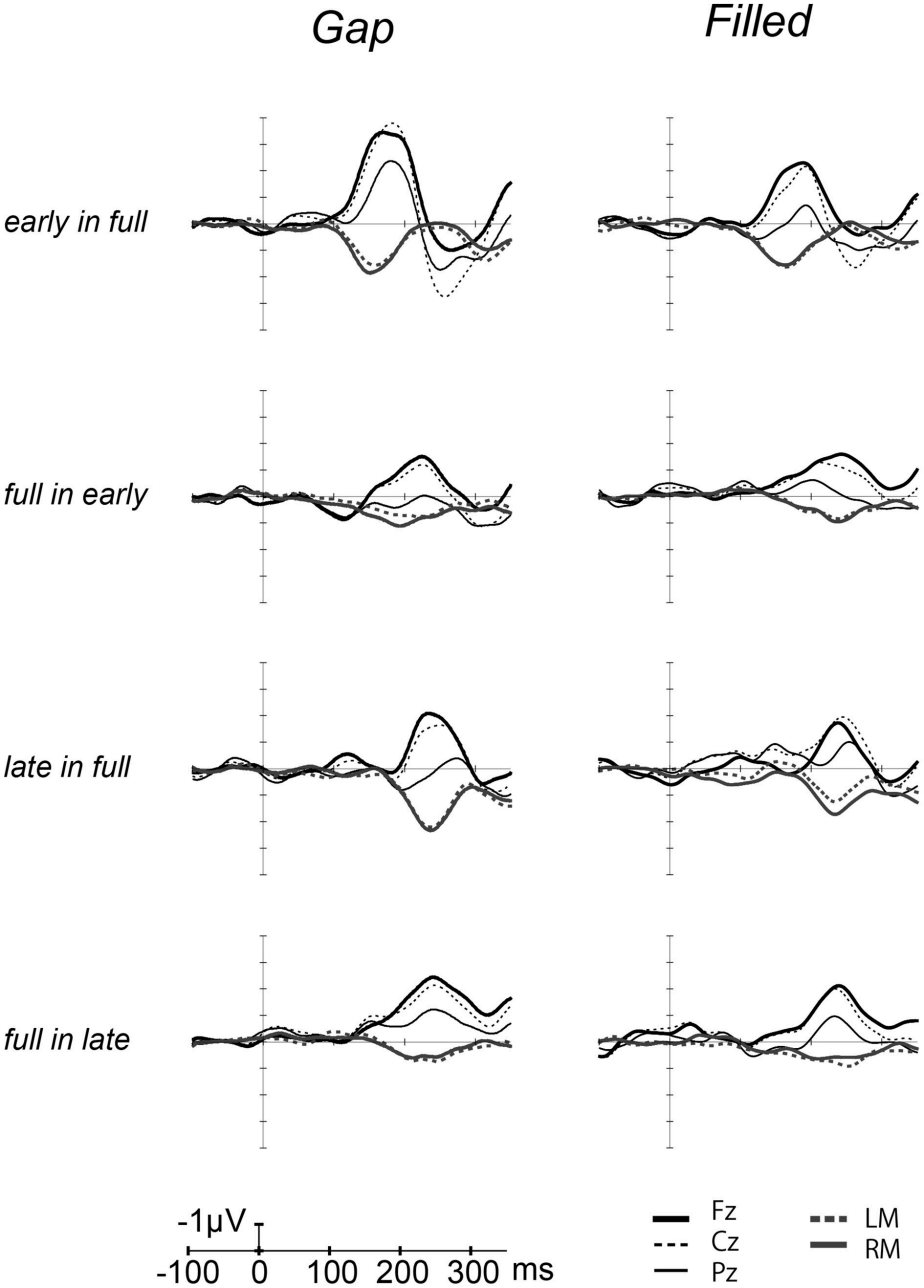
**Grand-averaged [deviant–standard] difference waves overlaid with each electrode**. The thick line waves indicate response from Fz, thin lines show the responses from Cz (dashed) and Pz (solid), gray lines recorded from LM (dashed) and RM (solid).

Figure 2 shows the ERP waves elicited by the standards and deviant stimuli for all oddball sequences. Negative deflections were observed at frontal sites, elicited by deviant stimuli in both gap and filled conditions. Figure 3 shows the [deviant—physically identical standard stimuli] difference waves for each condition. Visual inspection shows that inverted positive waves were observed at the mastoid electrodes, and the *t*-tests between the negative peak amplitude and zero showed significant differences in all conditions (*p*s < 0.05), indicating that the negative waves at Fz do represent MMN.

**Figure 4:**
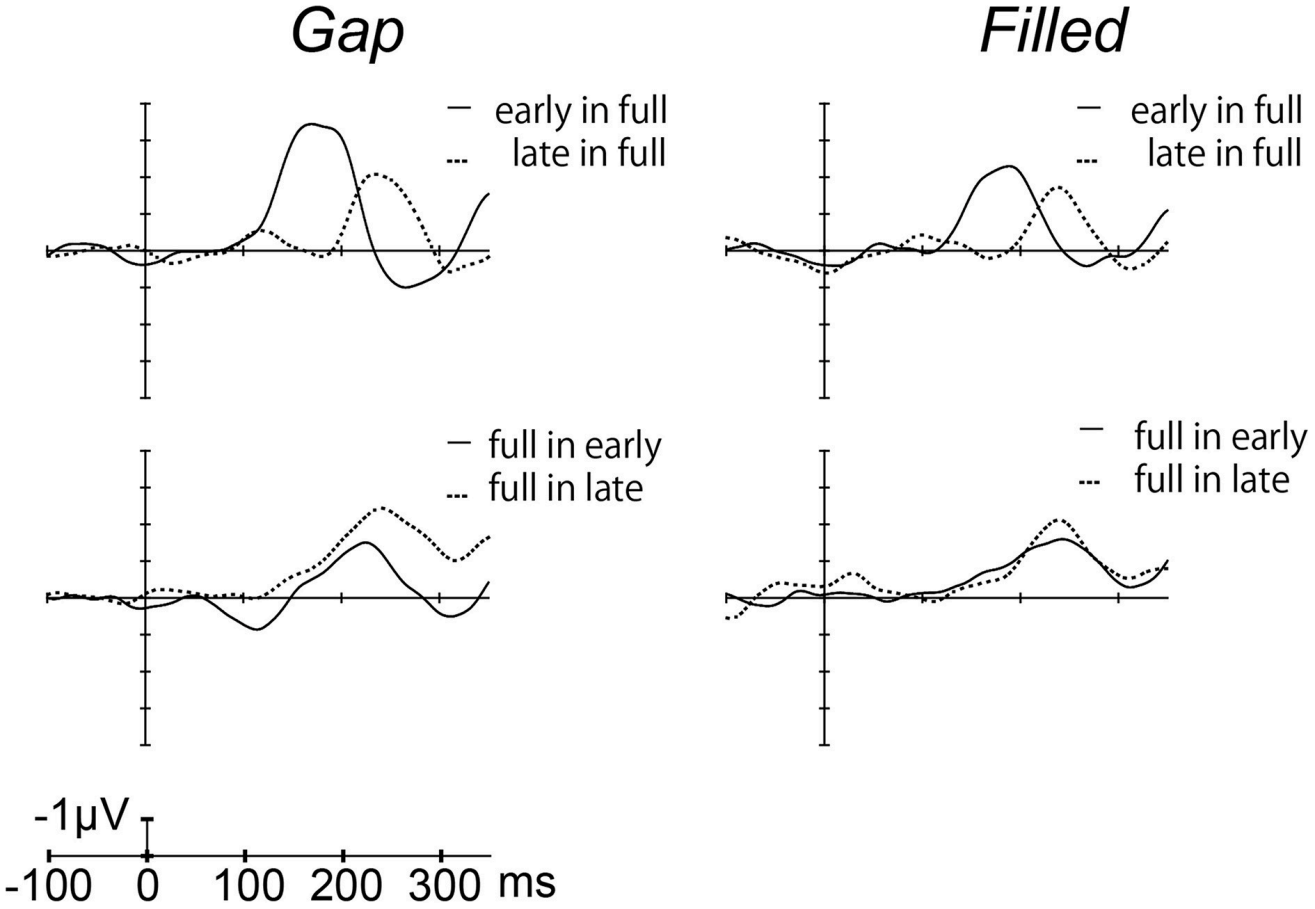
**Grand-averaged difference waves for the gap conditions (left panel) and filled conditions (right panel), in which ERPs to standard stimuli were subtracted from those to deviant stimuli at the Fz electrode**.

**Figure 5:**
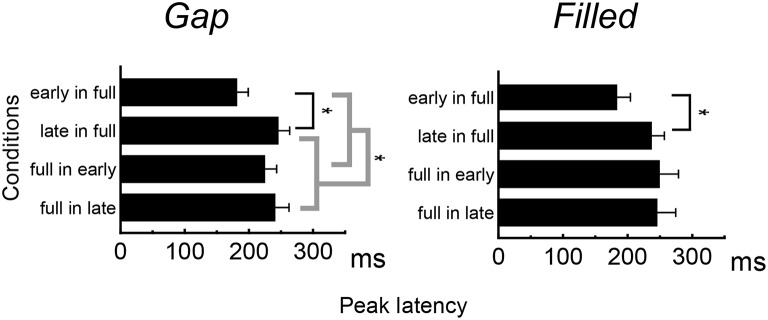
**Mean MMN peak latencies for deviant stimuli**. Error bars indicate standard deviations.

Figure 4 shows the difference waves at Fz, and indicating that MMN peaked at around 150–200 ms after stimulus onset. Peak MMN latencies varied as a function of gap timing or 1/4 segment (i.e., early vs. late) in deviants, under the condition where the standard was full-stimuli. In contrast, this effect of timing was not observed when the deviants were full-stimuli (Figure 5). Negative peak latencies were submitted to repeated measures ANOVA, with change type (gap vs. filled), timing (early vs. late), and sequence (in full vs. full in) as within-participants factors.

MMN peak latencies showed significant main effects of timing and sequence [timing: *F*_(1, 11)_ = 51.9, *p* < 0.05, η_*p*_^2^ = 0.83; sequence: *F*_(1, 11)_ = 27.0, *p* < 0.05, η_*p*_^2^ = 0.71]. The main effect of change type was not significant. There was a significant interaction between timing and sequence, *F*_(1, 11)_ = 20.4, *p* < 0.05, η_*p*_^2^ = 0.65. *Post hoc* analyses of simple main effects showed that MMN latencies for the early condition were significantly shorter than those for the late condition, but only when full-stimuli were standard. There was also a significant interaction between change type and timing, *F*_(1, 11)_ = 0.5.98, *p* < 0.05, η_*p*_^2^ = 0.35; a subsequent test of simple main effects showed that MMN latencies for the early condition were significantly shorter than those for the late condition when a gap was present. However, this simple main effect was not significant for the filled condition. There was no significant three-way interaction (Figure 5).

The same ANOVA was conducted for MMN peak amplitudes (Table 1). There was a significant interaction between timing and sequence, *F*_(1, 11)_ = 17.26, *p* < 0.05, η_*p*_^2^ = 0.61, with no significant main effect of change type (gap vs. filled). The MMN amplitudes for early conditions were significantly larger than those for the late conditions only when the full-stimuli were standard, for both gap and filled conditions.

**Table 1:** **Mean (standard deviation) MMN amplitude at Fz**.

	**Gap**	**Filled**
Early in full	−4.14 (2.58)	−3.01 (2.32)
Late in full	−2.53 (2.49)	−2.32 (2.20)
Full in early	−2.14 (2.87)	−2.32 (1.65)
Full in late	−2.98 (2.19)	−2.57 (2.42)
		(*N* = 12)

### Behavioral performance

RT data are displayed in Table 2. RTs were shorter in early conditions compared to late ones. The same ANOVA approach described above was used for RTs. There were significant main effects of change type and timing [change type: *F*_(1, 11)_ = 15.76, *p* < 0.05, η_*p*_^2^ = 0.59; timing: *F*_(1, 11)_ = 98.15, *p* < 0.05, η_*p*_^2^ = 0.90]. There was also a significant three-way interaction, *F*_(1, 11)_ = 5.97, *p* < 0.05, η_*p*_^2^ = 0.35.

**Table 2:** **Mean (standard deviation) reaction times and correct response rates in behavioral detection for each condition**.

	**Reaction time**		**Correct response rate (%)**	
	**Gap**	**Filled**	**Gap**	**Filled**
Early in full	326.4 (37.5)	349.56 (39.7)	88.0 (14.3)	84.1 (21.4)
Late in full	399.9 (24.7)	417.58 (22.2)	72.8 (27.8)	61.1 (30.9)
Full in early	359.9 (23.7)	356.14 (29.9)	88.7 (8.6)	84.8 (17.6)
Full in late	381.8 (19.5)	393.63 (24.7)	76.7 (20.8)	65.6 (24.9)
				(*N* = 12)

A subsequent ANOVA showed significant simple interactions between change type and sequence for the level of early, *F*_(1, 22)_ = 15.72, *p* < 0.05, η_*p*_^2^ = 0.42. *Post hoc* tests indicated the RTs for early in full were shorter than for full in early under the gap condition; however, these conditions did not differ for the filled condition.

Correct response rates (Table 2) were significantly lower in the filled than in the gap condition, *F*_(1, 11)_ = 8.26, *p* < 0.05, η_*p*_^2^ = 0.43. In addition, correct response rates for early stimuli were significantly higher than for late stimuli, *F*_(1, 11)_ = 19.2, *p* < 0.05, η_*p*_^2^ = 0.64.

## Discussion

MMN peak latency results show asymmetrical change not only in the gap conditions but also in the filled conditions, when reversing standard to deviant. As with a previous study (Yabe et al., 2005), MMN peak latencies showed a significant difference between early and late gaps in sequences presented with full-stimuli as the standard, whereas there was no such difference for sequences presented with full-stimuli as deviant. The same pattern of results was obtained for the filled conditions. The timing of the occurrence of deviation (i.e., the gap or weak segment in the filled stimulus) was the same regardless of the standard-deviant reversal. The present result of asymmetrical change in the MMN latencies clearly indicates that the temporal information contained in the neural representations and physical feature are not the same. In particular, because the latency difference between early and late was reduced when the full stimulus was deviant, Yabe et al. claimed that time is compressed in the sound representation. In the same manner, the present study shows that temporal information for the gap stimulus was certainly compressed in sensory memory representation. Furthermore, the same pattern for the filled stimulus condition showed that not the gaps but the whole stimulus duration was compressed.

One potentially concerning possibility is that because the intensity of the 1/4 filled stimulus was too weak, the filled and gap stimuli were not different in terms of registry on participants' auditory systems. However, this possibility can be ruled out by the significant interaction between change type and timing on MMN peak latency. In addition, correct response rates were lower and RTs were longer in the filled conditions compared to the gap condition. These results indicate that the filled stimuli were processed differently from the gap stimuli.

It seems that the mismatch process is likely to run two separate courses (Yabe et al., 2005). One of the processes is run under the real time-flow, whereas the other is processed according to rules of neural representation. When the incoming deviant stimuli feature some change information compared with standard stimuli, a matching process being carried out under the manner of real time-flow detects this change. When the repetitive sounds feature some acoustic change, temporal information regarding the change is maintained in imaginary in the neural representation. Such asymmetrical change is caused by time-compression in the neural representation.

RT results did not show the same pattern as the MMN latencies. This inconsistency was also observed in a previous study (Yabe et al., 2005). One possibility is that these discrepancies may represent a trade-off between accuracy and speed during processing, although the hit rate were not significantly different between early-in-full and full-in-early in each condition (gap and filled). The hit rate for full-in-late was lower than that for the other conditions; however, the MMN peak latencies were not delayed. These results suggest that the asymmetrical change in MMN latencies was not related to detection difficulty but rather to time compression in sensory memory.

The present study shows that time compression occurs for an entire sound, not for the silent portions within the duration of the TWI. The present results suggest that the silent gap was temporally maintained in auditory neural representation, as is the case for physical sound. This means that the human auditory system also encodes silence durations, as with a pause on a musical score. The pause or gap is also important information for verbal communication.

The TWI duration was estimated at about 170 ms in previous studies which used stimulus presentations that included a blank period between the two stimuli (e.g., tone pairs; Tervaniemi et al., 1994; stimulus omission occurring in constant SOA stimulus sequence: Yabe et al., 1997, 1999). These studies suggest that the silent parts of the stimulus within the TWI are also integrated into a unitary auditory object, and encoded as a part of the sound object *per se*. Because the stimulus duration was 176 ms in the present study, the effect occurred within the TWI, but it is not clear whether the same effect would occur outside of the TWI, which is an important topic. In addition, the auditory system extracts a sound from an acoustic context and integrates the sound within a neural representation. This hypothesis corresponds to a more recent interpretation of MMN regularity-violation, in terms of encoding regularity between sounds (Winkler et al., 2003; Winkler, 2007). Furthermore, the recent model of MMN and auditory processing reconfirmed the TWI as temporal and feature integration mechanism (Näätänen et al., 2011). Our finding that the time compression occurs in entire stimulus within the TWI suggests that the silence part is readily encoded in memory representation. These findings may support to further studies about time perception and neural activity (e.g., Van Wassenhove and Lecoutre, 2015).

Clarifying the nature of the time compression within the TWI would help develop our understanding of how automatic processing unfolds during listening in everyday life. For example, separation or connection of phonemes that are inputted in proximity are essential for word recognition. It is thought that temporal integration plays an important role in the process to connect such phonemes. Temporal integration is likely to be reasonably flexible, such that encoding of silence can still play an important role.

## Conclusion

Asymmetrical change in MMN peak latencies was observed using stimuli that included not only silent gaps but also intensity changes. This means that time compression occurs for an entire stimulus representation within the temporal window of integration. The pattern suggests that the temporal aspect of silence is processed in the same way as sound in neural representation.

## Author contributions

ST, NM, JK, and AY contribute to the designing of the experiment, and analyses and interpretations of the data, drafting the article. Especially, NM acquired the data, JK, and AY revised manuscript critically. ST, NM, JK, and AY approve to the publication, and agree to be accountable for all aspects of the work.

### Conflict of interest statement

The authors declare that the research was conducted in the absence of any commercial or financial relationships that could be construed as a potential conflict of interest.
